# Controversies in Venous Thromboembolism Risk Assessment in Inflammatory Bowel Disease: A Narrative Review

**DOI:** 10.3390/diagnostics14192112

**Published:** 2024-09-24

**Authors:** Nikhil Sharma, Pavit Tewatia, Philip R. Harvey, Aditi Kumar

**Affiliations:** Department of Gastroenterology, The Royal Wolverhampton NHS Trust, Wolverhampton WV10 0QP, UK; nikhil.sharma11@nhs.net (N.S.); pavit.tewatia@nhs.net (P.T.); aditikumar@nhs.net (A.K.)

**Keywords:** venous thromboembolism, inflammatory bowel disease, ulcerative colitis, Crohn’s disease, pregnancy

## Abstract

Inflammatory bowel disease (IBD) is a chronic inflammatory condition affecting the gastrointestinal tract with increasing rates of incidence and prevalence across the world. Complex inflammatory and prothrombotic pathophysiology in IBD makes venous thromboembolism (VTE) a common complication with significant morbidity and mortality. This risk is increased in pregnancy. As we continue to understand the pathogenesis of IBD, this article highlights the continued risk of VTE following discharge, for which there is currently no clear guidance, yet the risk of VTE remains high. Furthermore, we discuss this increased VTE risk in the context of pregnant IBD patients and the relevant current guidelines. Alongside this, medications that are used to manage IBD carry their own thrombotic risk, which clinicians should be aware of. Assessing VTE risks in IBD populations using newer medications should be a focus of future research.

## 1. Introduction

Inflammatory bowel disease (IBD) comprises ulcerative colitis (UC) and Crohn’s disease (CD) and is a group of chronic, relapsing–remitting diseases that cause inflammation of the gastrointestinal tract [1]. IBD can affect individuals of all ages, gender, ethnicities, and socioeconomic parameters and has a rising worldwide incidence and prevalence, with the highest rates reported in the Western nations [1,2]. The pathogenesis of IBD is multifactorial, including genetic factors, environmental factors and gut microbiome, which leads to a persistently active immune response causing inflammation in the gut. The chronic nature of this disease demands continuous secondary care input, requiring repeat invasive investigations, hospital admissions, and oftentimes surgical input. There is an increasing demand for the initiation and maintenance of this disease with expensive advanced therapies such as immunosuppressive medications.

Whilst IBD predominantly affects the small and large bowel, it can also be associated with numerous extraintestinal complications such as inflammatory arthritis, uveitis, pyoderma gangrenosum, erythema nodosum, primary biliary cirrhosis, and primary sclerosing cholangitis. Among these complications is the risk of venous thromboembolism (VTE), which is associated with significant morbidity and mortality, and incurs a considerable health burden to patients with IBD [3,4]. This review article will provide an overview of the pathophysiology of VTE in IBD and will discuss the general risk factors before focusing on the prophylaxis and management for patients in three specific scenarios: pregnancy, following hospitalisation, and usage of the medical therapies in IBD.

## 2. Epidemiology

Compared to the general population, patients with IBD have a two- to threefold increased risk of developing deep venous thrombosis (DVT) and pulmonary embolism (PE) [5,6,7,8,9], with a global prevalence that surpasses 0.3% [2]. The most important risk factors for developing VTE are active disease, hospitalisation, and surgery [10]. Interestingly, whilst patients with IBD had the highest risk of VTE at the time of disease flare-up (hazard ratio (HR) 8.4 compared to control group), there was also an increased risk during disease remission (HR 2.1 compared to control group). This suggests an overall procoagulant tendency in IBD [8], which may be a combination of both hereditary and acquired factors [11].

Pregnant women have been reported to develop VTE four to five times more frequently than nonpregnant women [12,13]. Kim et al. proposed that a synergistic interaction exists between IBD and pregnancy for VTE which results in a 10-fold increase in risk, when compared to nonpregnant women without IBD [14]. Increased risk of VTE has been postulated to persist for 12 weeks postpartum due to the physiological changes of pregnancy including alterations in venous blood flow and vascular injury [15].

## 3. Pathophysiology and Risk Factors for VTE in IBD

There is evidence for genetic predisposition in pathogenesis of IBD. Genes like nucleotide-binding oligomerization domain-containing protein 2 (NOD2), recombination activating gene 2 (RAG2), interleukin 10 (IL-10) receptor deficiency, the autophagy gene (ATG16L1), and nuclear factor kappa beta (NF-kB) essential modulator (NEMO) have all been associated with IBD [16]. Mutations in NOD2, seen in mice, have shown significant alteration in gut microbiota [17], leading to a decrease in the anti-inflammatory bacterium, *Faecalibacterium,* and an increase in the infectious bacterium, *Escherichia.* NOD2 mutation also leads to defective phagocytosis of bacteria and compensatory heightened immune response resulting in inflammation [18]. Inflammatory cytokines such as interleukin 1, 6 and 8 (IL-1, IL-6 and IL-8) along with tumour necrosis factor (TNF) activate tissue factor (TF), thereby activating the coagulation cascade [19]. Thrombosis in IBD involves abnormal regulation of coagulation activity, disturbance of fibrinolysis, inflammatory reactions, and thrombocytosis. A clear relationship has been seen between inflammatory and coagulation pathways. Inflammatory cytokines activate signalling pathways, which induce a hypercoagulable state and promote the formation of thrombi by inhibiting natural anticoagulant and fibrinolytic systems [20]. Platelets are an important component of the coagulation cascade and, once activated, are known to release a range of inflammatory mediators (platelet activating factor (PAF), thromboxane A2 (TXA2), platelet factor 4 (PF4), and transforming growth factor-β (TGFβ)). This then helps to recruit inflammatory cells (neutrophils, monocytes, and eosinophils) and modulate the activity of these inflammatory cells [21].

**Figure 1:**
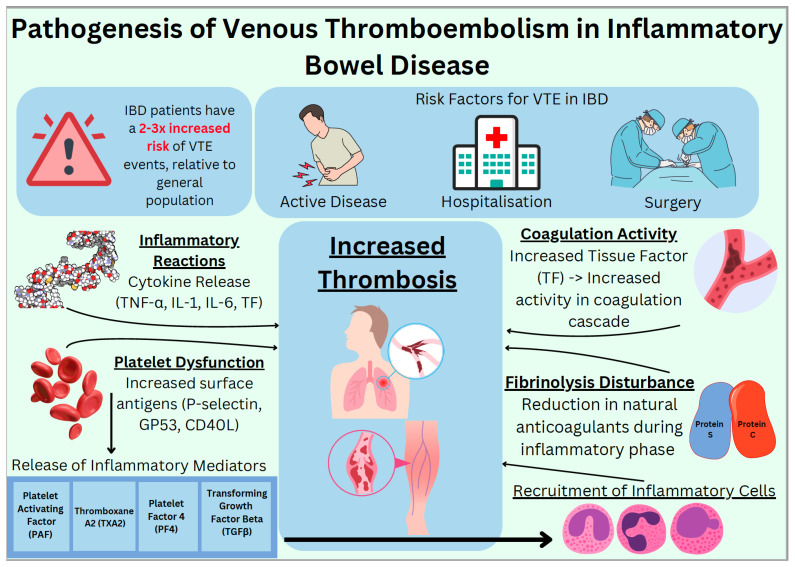
The main recognised risk factors for VTE events, and processes that increase the risk of thrombosis in patients with IBD.

In patients with IBD, there is clear evidence of thrombocytosis [22], but the cause remains unclear. However, we know that there is platelet dysfunction which leads to increased expression of activation dependent surface antigens on platelets (P-selectin, GP53 and CD40 Ligand (CD40L)). Raised plasma levels of CD40L are also known to be positively correlated with prothrombotic processes in IBD [23]. Inflammatory response and activation of coagulation activity complement each other in IBD. Chronic inflammation mass produces proinflammatory cytokines like Tumour Necrosis Factor-Alpha (TNF-α) and interleukins (IL-1 and IL-6) and increases the production of both tissue factor (TF) and the coagulation cascade. The aggregation of platelets and inflammatory response predispose to thrombus formation. During the active inflammatory phase, there is a decrease in the natural anticoagulants such as protein C and protein S, which are not able to counteract this excess thrombus formation [24]. Figure 1 below shows a simplified schematic on the pathophysiology mentioned.

The increased risk of VTE during a flare-up in IBD for hospitalised patients is explained by the above mechanism. This prothrombotic inflammatory process takes time to settle over time following discharge; therefore, VTE risk remains high, even after discharge.

VTE is predominantly seen in the deeper leg and pulmonary veins, but can also involve mesenteric, portal, retinal, and cerebral veins [25,26,27]. Papay et al. observed that 9.6% of their IBD study group had different locations of VTE than the commonly found locations, i.e., cerebral, portal, mesenteric, internal jugular, and splenic veins [28]. One could argue that as the inflammation is predominantly in the bowel, abdominal vessels are more prone to developing VTE, especially in the portal vein [29]. This highlights the risk of different locations of VTE in patients with IBD and the need for prophylactic treatment during a flare-up and potential need following discharge.

Patients with UC have generally been observed to have higher risk of developing VTE than CD. Saleh et al. compared UC and CD and found that UC had a higher risk of PE, DVT, and VTE (relative risk (RR) of 1.92, 1.43, and 1.52, respectively) [30]. Bernstein et al. also observed an increased risk of VTE in IBD than the general population (two- to threefold) but noticed that patients with CD were more prone to developing DVT than patients with UC (CD incidence 31.4/10,000 person-years vs. UC 30.8/10,000 person-years). In Bernstein’s study, UC patients were more prone to developing PE than CD (UC incidence 19.8/10,000 person-years versus CD incidence 10.3/10,000 person-years) [10]. The cause for these findings is unclear, and further research is needed to identify the reasoning for this perceived increased risk.

The time of diagnosis for IBD has a bimodal distribution, with an initial peak between adolescence to mid-30s. The second peak tends to be seen in patients in their 60s. Studies have demonstrated a variable impact of age at diagnosis. Papay et al. noted that patients who developed VTE did so if they were slightly older at the time of IBD diagnosis compared to those who did not (34.4 ± 14.8 years vs. 32.1 ± 14.4 years, *p* = 0.045) [28].

In comparison, Kappelman et al. noted the risk of VTE in IBD in a Danish population to be higher in patients aged 20 and below (HR 6.0 for DVT and HR 6.3 for PE). The risk was also higher for total events (HR 2.0 (95% CI 1.8 to 2.1)) and for unprovoked events (HR = 1.6 (95% CI 1.5–1.8)) [9]. Age at diagnosis, therefore, requires further investigation regarding the impact on risk of VTE in IBD patients.

Interestingly, whilst an association between VTE and IBD is well described, a similar association is not observed with other autoimmune conditions such as coeliac disease, rheumatoid arthritis, and other chronic inflammatory conditions. This was demonstrated by Meihsler et al., reporting a statistically significant 3.6-fold higher risk of VTE in IBD patients compared to age- and sex-matched controls [31]. ORs for rheumatoid arthritis and coeliac disease were statistically insignificant at 0.7 and 0.4, respectively, relative to age- and sex-matched controls. The reason behind these findings is unclear. Further studies comparing autoimmune conditions with IBD are required to identify whether VTE risk is specific to IBD.

Another common comorbidity reported amongst IBD populations is diabetes mellitus, both type 1 (T1DM) [32] and type 2 (T2DM) [33]. Whilst the prevalence of diabetes mellitus is well established with IBD, the effect on pathogenesis of thrombosis in IBD has not yet been extensively explored. In one retrospective cohort study, Achebe et al. investigated the thrombotic predictors in IBD and colon cancer cohorts. One variable explored as a thrombotic predictor in this study was uncomplicated diabetes mellitus. Interestingly, patients with uncomplicated diabetes seemingly had lower rates of VTE relative to those without diabetes (OR 0.48, 0.28–0.84, *p* < 0.010), implying a protective factor against VTE events [34]. However, complicated diabetes in this study demonstrated a statistically insignificant result. A meta-analysis exploring the influence of diabetes mellitus on IBD concluded that whilst diabetes may affect the course of IBD, there was no increased risk in IBD-related complications (such as intraabdominal abscesses, stricturing disease, and toxic megacolon) and mortality [35]. This meta-analysis, however, did not comment directly on VTE risk. The relationship of the effect of diabetes on IBD and VTE risk needs further attention, with focused studies examining VTE risk in IBD populations. Furthermore, additional studies delineating the effect of complicated and uncomplicated diabetes mellitus upon VTE incidence are needed.

## 4. Pregnancy

The timeline of VTE risk prevalence during pregnancy is conflicted in the literature, and studies suggest higher incidence in the latter trimesters [36,37,38], while other studies indicate similar (but elevated) incidence levels across all three trimesters [39,40,41,42]. Conclusions stating VTE risk being highest in the postpartum period are more concrete [43], with the peak incidence in the first 3–6 weeks [44,45].

IBD and pregnancy itself is a challenging area, and the risk of VTE becomes much higher in pregnancy. Nguyen et al. noted an increased VTE risk in pregnancy, which was substantially higher in IBD (adjusted odds ratio (CD (aOR) 6.12; 95% CI: 2.91–12.9 and UC (aOR) 8.44; 95% CI: 3.71–19.2) than non-IBD patients [46]. Kim et al. analysed subgroups of patients with UC and CD, which demonstrated higher VTE risk in UC patients both in the antenatal and postpartum periods (RR = 2.24 and 2.85 when comparing antenatal UC vs. CD, and postpartum UC vs. CD) [14]. This highlights that UC is more prone to having VTE in pregnancy. The current literature estimates an eightfold increase in VTE risk in IBD flare-ups in pregnant women; however, this figure is derived from studies with results of statistical insignificance and high degree of heterogeneity [47,48,49].

The Royal College of Obstetricians and Gynaecologists (RCOG) [50], British Society of Gastroenterology (BSG) [51], and European Crohn’s and Colitis Organisation (ECCO) [52] recommend that all patients with IBD who become pregnant should be assessed for VTE risk as early as possible with a formal risk assessment (see Supplementary Materials for RCOG adaptation), with careful reassessment following any hospital admissions, intercurrent problems, and immediately following delivery.

The RCOG have provided guidance for the use of low-molecular-weight heparin (LMWH) in pregnancy. Any history of a previous, unprovoked VTE is considered as high risk, and RCOG recommends the use of LMWH antenatally and for six weeks postnatally in these patients, regardless of IBD diagnosis. IBD and other prothrombotic states such as systemic lupus erythematosus (SLE) are considered as intermediate risk and RCOG suggests considering the use of LMWH antenatally and for 10 days postnatally [50]. Table S2 in the Supplementary Materials is an adaptation from RCOG regarding risk factors of VTE in pregnancy including IBD and the corresponding thromboprophylaxis recommendation based on corresponding risk stratification.

The guideline from ECCO also recommends the evaluation of this risk as early as possible and the use of prophylactic LMWH in IBD flare-ups requiring hospital admission. For active disease, ECCO suggests that outpatients with moderate or severe IBD flare-up and a high-risk VTE profile may benefit from pharmacological thromboprophylaxis until the resolution of the flare-up [52].

The combination of pregnancy and IBD results in a synergistic, prothrombotic state where thromboprophylaxis and regular VTE assessments are required.

## 5. Post-Discharge Prophylaxis

VTE prophylaxis is a well-recognised treatment that is mandated for all IBD patients admitted into hospital, regardless of the reason for admission [24,46]. National and international guidelines such as those produced by BSG, ECCO, and the American Society of Gastroenterology all recommend the use of prophylactic LMWH whilst admitted into hospital [52,53,54]. These patients, however, are not only predisposed to VTE during a flare-up but also following hospital discharge, with the risk of VTE being maintained up to 90 days following discharge [55].

Faye et al. evaluated readmissions with VTE following discharges and found that the risk was highest in the first ten days. Factors associated with increased risk for VTE whilst an inpatient included having a flexible sigmoidoscopy or colonoscopy on index admission, *Clostridioides difficile* infection, and being discharged to a nursing or intermediate facility. Both UC and CD had similar rates of readmission with VTE, and patients who were readmitted had a 50% higher mean length of stay [56]. It is hypothesised that although patients are discharged from hospital once deemed medically stable, the inflammation in the gut takes time to settle and, therefore, the additional VTE risk does take time to return to the patient’s baseline. Harvey et al. found that the rate of VTE following any admission (emergency admission with or without surgery, elective admission with surgery) was 17.2 per 1000 patient years [55]. Similar findings were seen by Grainge et al., where they found that at the time of a flare-up, the risk of developing VTE was 9.0 per 1000 person-years (8.4, 5.5–12.8, *p* < 0.0001). The cohort of patients evaluated had a higher relative risk for VTE during nonhospitalised periods (15.8, 9.8–25.5; *p* < 0.0001) than during their hospital stay (3.2, 1.7–6.3; *p* = 0.0006) [8]. It is hypothesised that the higher observed risk in nonhospitalised patients pertains to the VTE prophylaxis routinely provided when admitted to hospital. These figures highlight the importance of recognising the risk of VTE post-discharge.

Chu et al. also evaluated the post-discharge VTE risk in IBD patients with or without surgery. They found that the risk of developing VTE in patients not undergoing major surgery was lower (HR 1.13 (0.63–2.02)) when compared to patients undergoing major IBD surgery (2.43 (1.20–4.92)). IBD patients undergoing surgery had elevated risk for developing VTE up to 6 weeks following surgery (absolute risk 18.6/1000 person-years) [57]. McKechnie et al. also found an increased postoperative risk of VTE in IBD patients who underwent colorectal surgery, especially for UC [58]. Ore et al. conducted a large retrospective analysis of IBD patients undergoing surgery and found that the median time for VTE post-surgery was 9 days. They also found that patients undergoing complex procedures had an increased rate of VTE (3.6%; 361/10,178) [59]. IBD patients undergoing surgery have a higher risk postoperatively of VTE and subsequent increased risk of morbidity and mortality.

VTE in patients with IBD following hospital admission is a challenging area to study. There is clear evidence of ongoing risk following discharge in both patients admitted for active disease and patients undergoing IBD surgery. Presently, there are no studies to inform clinicians of the overall risks and benefits that VTE prophylaxis would deliver in this patient group. The recent international guidelines for the prevention of VTE in IBD state that an extended thromboprophylaxis course can be considered for an additional 2–8 weeks following hospital discharge in patients with a high risk for VTE; however, there is no strict criteria to help identify these high-risk patients [60]. Thus, further research is needed in this area.

## 6. Medication

There is a variable risk of VTE associated with several common medications used for treatment of IBD. VTE risk in IBD patients is variable depending on disease phenotype, such as UC vs. CD, and the presence or absence of active inflammation. Therefore, delineating the degree of additional risk from medications used for treating IBD, rather than the additional risk conferred by IBD at a given time point, is often challenging. The mechanisms by which medications confer additional risk vary, including their effect on homeostasis, particularly in inducing a prothrombotic versus a hypercoagulable state [61]. VTE risk associated with medications, in addition to the prothrombotic nature of IBD, should be of clinical note and concern to physicians. In this section, we discuss the mainstays of IBD therapy and their literature-reported thrombotic effects in IBD populations (if available). The perceived thrombotic effect in IBD populations is described in Table 1.

### 6.1. 5-ASAs (5-Aminosalicylic Acid)

The 5-aminosalicylic acids (5-ASAs) exert their therapeutic effects in IBD through multiple mechanisms, including the inhibition of proinflammatory cytokines such as TNF-a and interleukins, suppression of leukocyte chemotaxis, and scavenging of free radicals. These actions collectively help to mitigate inflammation in the intestinal mucosa, thereby alleviating symptoms and promoting mucosal healing [62]. Specifically designed studies are not available in determining the VTE risk of 5-ASAs in an IBD population; however, an available, in vivo study of 5-ASAs suggests an antithrombotic effect [63].

### 6.2. Corticosteroids

Corticosteroids in IBD primarily function by modulating immune responses through various mechanisms, such as inhibition of proinflammatory cytokines and suppression of T-cell activation [64]. These actions lead to the alleviation of inflammation and symptom improvement.

Despite being used as an anti-inflammatory agent, corticosteroids have been widely documented to induce a prothrombotic state. In a trial involving healthy populations taking oral prednisolone (0.5 mg/kg/day) or a placebo for 10 days, prednisolone intake was associated with increased peak thrombin levels, plasminogen-activator inhibitor type-1, and von Willebrand factor (vWf) levels relative to placebo [65]. In a meta-analysis comparing six studies assessing VTE risk in CD and UC patients treated and not treated with corticosteroids, there was a significantly higher rate of VTE complications in steroid-treated IBD patients (OR: 2.202, *p* < 0.001) [66]. The current literature of corticosteroids being used in an IBD population demonstrates a prothrombotic picture.

### 6.3. Thiopurines

In IBD, the action of thiopurines is to inhibit DNA and RNA synthesis through the inhibition of purine metabolism, which acts on dampening the response of immune cells within the gastrointestinal tract. This leads to decreased lymphocyte proliferation and modulation of the inflammatory response within the gastrointestinal tract, aiding in the induction and maintenance of remission in IBD [67]. Azathioprine also inhibits the formation of platelet–leukocyte aggregates and platelet aggregation in vitro [68].

In a study looking at VTE risk in Asian patients with IBD, a hazard ratio of 0.53 (0.39–0.71, *p* < 0.001) was reported for patients taking thiopurines (*n* = 14,866). Subgroup analysis of this study demonstrated similar hazard ratios for CD and UC patients (0.47 (0.29–0.77, *p* = 0.002) and 0.59 (0.41–0.85, *p* = 0.004) respectively) [69].

Evidence from the literature relating to immunomodulators in IBD and their VTE risk is limited. Current evidence suggests that thiopurines have little effect on VTE risk in IBD groups, but this needs further research confirmation.

### 6.4. Methotrexate

The mechanism of action for methotrexate involves its ability to inhibit dihydrofolate reductase (DFT), leading to the suppression of DNA synthesis and cell proliferation, particularly in rapidly dividing immune cells. This immunosuppressive effect contributes to the reduction in inflammation [70].

Contrastingly, elevated serum levels of homocysteine as a byproduct from the inhibition of DFT has been shown to be associated with increased VTE incidence; however, the mechanism for this is not fully known. In one study, IBD patients have been reported to also have a higher prevalence of hyperhomocysteinemia, with folate deficiency being the only independent risk factor for this development [71]. To combat the prothrombotic nature of methotrexate, concurrent oral folic acid is advised.

There is currently no evidence in the literature that links the use of methotrexate with VTE risk in patients with IBD. In patients with rheumatoid arthritis, however, evidence suggests that methotrexate has the lowest incidence level for VTE risk (3.5/1000 person-years) compared to disease-modifying antirheumatic drugs (DMARDs, 5.5/1000 person-years) and other nonbiologic DMARDs (4.4/1000 person-years) [72].

The risk of VTE in methotrexate has only been assessed in IBD in the context of following failed thiopurine therapy in CD, as either monotherapy or dual therapy. Long-term use (*n* = 259, over 5 years) of methotrexate use yielded no VTE events, and, thus far, has been concluded to be safe [73].

### 6.5. Tumour Necrosis Factor-Alpha Inhibitors (TNF-α)

TNF-a promotes inflammation by activating immune cells and inducing production of proinflammatory cytokines. This overexpression of cytokines leads to chronic inflammation, and mucosal damage in the gastrointestinal tract [74]. TNF is also known to be involved in the activation of the coagulation pathway; thus, it is thought that anti-TNF medications, in addition to their anti-inflammatory actions, could also help prevent the formation of thrombi [75]. Infliximab (IFX) has been reported to decrease or normalise clot lysis profiles [76] in a prospective study comparing infliximab, vedolizumab, and methylprednisolone, with Bollen et al. suggesting that the initiation of IFX in IBD patients can normalise deranged haemostatic profiles [77].

Numerous studies have demonstrated no increased risk of VTE with anti-TNFα agents. A retrospective analysis (*n* = 15,100) involving IBD patients was undertaken to assess the impact of biologic therapies (all of which were TNF-α agents), corticosteroids, and their combination on the risk of VTE over 12 months of follow-up. The study concluded an odds ratio of 0.21 (0.05–0.87, *p* < 0.05) when comparing biologic monotherapy to corticosteroid therapy. Importantly, the combination therapy resulted in a VTE frequency risk similar to corticosteroid monotherapy (2.49% (1.76–3.42) vs. 2.25% (2.00–2.52), *p* = 0.028), suggesting corticosteroids to be the primary contributor to this VTE risk [78]. A further meta-analysis further demonstrated a fivefold decreased risk of VTE with anti-TNFα agents compared to corticosteroids (OR 0.267, 95% CI 0.106–0.674, *p* = 0.005) [66]. Another study by De Fonseka et al. on VTE events in IBD patients supports an antithrombotic property of TNF-α inhibitors with an OR of 0.20 (*p* = 0.049), as well as systemic corticosteroid prothrombotic action with an OR of 4.62 (*p* = 0.0004) [79].

### 6.6. Janus Kinase Inhibitors (JAKi)

Janus kinases (JAK) are involved in signalling pathways of various proinflammatory cytokines [80], and the inhibition of those cytokines suppresses inflammation and provides symptomatic relief for IBD patients.

A recent meta-analysis by Zhang et al. explored VTE risk in four JAKi inhibitors at different doses. In this meta-analysis, the JAKi and corresponding doses were as follows: upadacitinib (7.5 mg, 15 mg, 30 mg, and 45 mg once daily (OD)), tofacitinib (5 mg and 10 mg twice daily), filgotinib (100 mg and 200 mg OD), and baricitinib (2 mg and 4 mg OD). The 16 studies (*n* = 17,242) used in this meta-analysis were all phase 2 or 3 randomised controlled trials (RCTs) with a JAK inhibitor therapy with at least a placebo comparator arm, with some studies including a TNF-α comparator group alongside. The overall pooled risk ratio (RR) for JAKi versus placebo group was 0.72 (95% CI 0.33, 1.55: *p* = 0.40), and a pooled RR for JAKi versus TNF-α was 0.94 (95% CI 0.33, 2.69: *p* = 0.91).

Of all the four JAKi therapies relative to placebo, filgotinib demonstrated the lowest RR of 0.14 (95% CI 0.03, 0.74: *p* = 0.02) whilst baricitinib exhibited the highest RR of 1.51 (95% CI 0.06, 36.86: *p* = 0.80). Comparing JAKi to TNF-α therapy, upadacitinib had the lowest RR of 0.50 (0.19, 1.31: *p* = 0.16), with Tofacitinib demonstrating the highest RR of 2.54 (1.29, 4.99: *p* = 0.007). Subgroup analysis also showed lower rates of VTE events with lower doses of JAKi therapy (RR = 0.56, 95% CI (0.36–0.88): *p* = 0.01). Overall, the incidence of VTE was not significantly higher with JAKi, compared to TNF-α inhibitors and placebo [81]. These conclusions were in line with previous meta-analysis on JAKi and VTE risks [82,83]. The RCTs used in the meta-analysis, however, were not IBD-exclusive patients, and included immune-mediated inflammatory diseases (IMIDs) such as rheumatoid arthritis, asthma, and autoimmune neurological diseases.

In a meta-analysis involving a UC population with 17 studies (*n* = 1162) focusing on the real-world effectiveness and safety of tofacitinib, safety outcomes were reported in 15 of those studies (*n* = 972). Of those 15 studies, no major thromboembolic complications were reported [84].

One phase 3b-4 safety endpoint trial (Oral Rheumatoid Arthritis Trial (ORAL surveillance)) focusing on cardiovascular and cancer risk with tofacitinib, where patients were assigned in a 1:1:1 ratio to receive tofacitinib 5 mg BD (*n* = 1455) or 10 mg BD (*n* = 1456) or a TNF-α (*n* = 1451) inhibitor, reported VTE, including DVT and PEs as secondary safety endpoints. Adjudicated VTE events were more frequent, with tofacitinib at 10 mg BD, compared to TNF- α inhibitor (HR 3.52 (1.74–7.12)). Adjudicated PE and DVT HRs were also greatest at the higher dose of 10 mg Tofacitinib BD compared to 5 mg BD (8.26 vs. 2.93, and 2.21 vs. 1.54, respectively). However, the application of this trial in an IBD context is ill-fit. The ORAL surveillance study only included patients with active rheumatoid arthritis who were 50 years of age or older, whereas IBD patients tend to initially present earlier as part of the bimodal distribution [85].

In another post hoc analysis of Tofacitinib in an exclusive UC cohort (*n* = 1157), they reported five VTE cases (one DVT case and four PE cases) in patients treated with 10 mg BD, and four cases in placebo-treated patients [86]. These five cases of the Tofacitinib 10 mg BD subgroup had pre-existing risk factors for VTE such as active disease, cardiovascular history, and malignancy.

A limitation with this study is its limited sample size, and further research in an IBD population for Tofacitinib is needed. Specific studies evaluating VTE risk with Tofacitinib at higher doses in greater sample sizes are necessary to provide clinicians with an evidence base on which they can formulate individualised decision making on treatment initiation, escalation, and de-escalation of doses.

In three phase 3 trials (U-EXCEL (*n* = 526), U-EXCEED (*n* = 495), and U-ENDURE (*n* = 502)) analysing the effectiveness of Upadacitinib as induction and maintenance therapy for CD, safety outcomes including thromboembolic events were assessed by independent adjudication committees. Across the three trials, only one thromboembolic event was recorded, which was a hepatic vein thrombosis concurrent with exacerbation of CD in a patient taking Upadacitinib 30 mg [87]. Due to the context of the single VTE event and overall sample size, a conclusion regarding Upadacitinib and dosage-related VTE risk cannot be made with much certainty.

In a phase 2b study (*n* = 250) focusing on the efficacy and safety of Upadacitinib as induction therapy for UC, only one patient had developed PE and DVT, which was diagnosed 26 days after the drug had been discontinued and was in an episode of UC flare-up [88]. In this study, patients received varying doses of Upadacitinib such as 7.5 mg, 15 mg, 30 mg, or 45 mg. The patient with the VTE event received Upadacitinib 45 mg, with risk factors such as previous smoking, hospitalisation, active disease, and concomitant use of corticosteroids. It is inconclusive whether this VTE event was a result of Upadacitinib, disease progression, individualised risk factors, or a combination of factors. Like Tofacitinib, studies exploring VTE risk at differing doses of Upadacitinib are required for clinicians to make an informed decision regarding the overall benefit of this treatment.

At the time of writing, IBD teams are generally cautious with respect to JAKi and their risk of VTE. However, the evidence base for VTE risk in JAKi specific to the IBD population is lacking and remains a controversial topic that warrants further research. Currently, the European Medicines Agency and the American Food and Drug Administration recommend the use of JAKi agents only if no suitable treatment alternatives are available in patients above the age of 65, in patients with an increased risk of major cardiovascular events or cancer, and in patients who are currently smoking.

### 6.7. Sphingosine 1-Phosphate (S1P) Receptor Modulators

The pathogenesis of IBD involves the migration of lymphocytes from lymphoid tissues to the intestines, where they contribute to inflammation. S1P signalling regulates lymphocyte trafficking and promotes inflammation by binding to S1P1 receptors, facilitating lymphocyte migration into circulation and inflammatory sites [89].

In a study (*n* = 823) looking at cardiac and vascular treatment-emergent adverse effects of the primarily used S1P in UC, Ozanimod, the incidence for DVT and PE was low at 0.2% for both. The low incidence of VTE was consistent in both the induction and maintenance period of Ozanimod treatment [90]. The Phase 2 TOUCHSTONE study (*n* = 197) is consistent with this, as no VTE events were reported in over 4 years of follow-up in patients with moderate to severe UC treated with Ozanimod [91].

### 6.8. Anti-Integrin Therapy

Anti-integrins in IBD work by blocking the interaction between integrins (such as α4β7) expressed by circulating immune cells and their ligands (like MAdCAM-1) on endothelial cells, thereby preventing the migration of inflammatory cells into the gut tissue and reducing inflammation [92]. Evidence for anti-integrin VTE risk in IBD is limited. Four reports (*n* = 2830, *n* = 321, *n* = 136, *n* = 132) focusing on the safety profile and treatment efficacy of Vedolizumab did not explicitly report any VTE events in IBD populations [93,94,95,96].

In contrast, an abstract from Facey et al. reported long-term risk of VTE in CD patients treated with biologics or immunomodulators, including anti-integrin therapy, Anti-TNFa, Anti IL-12, and 23 inhibitors. Between 3 months and 5 years of medication initiation, anti-integrin therapy (*n* = 23,384) had the second highest rates of DVT and the highest rate of PE at 6.7% and 5.0%, with immunomodulators attaining 7.1% and 4.9%, respectively [97].

Currently, the literature regarding VTE risk in anti-integrin therapy is limited in the long term, with no clear conclusion on the risk of VTE in its use, and should be a focus for further research.

### 6.9. Anti IL-12 and Anti IL-23

Interleukin (IL)-12 and IL-23 are proinflammatory cytokines involved in the activation of Th1 and Th17 cells leading to gut inflammation. Disruption through blocking activity of these cytokines leads to a decreased inflammatory response [98]. Ustekinumab is a human immunoglobin (Ig) G1 kappa monoclonal antibody directed against IL-12 and IL-23. This drug has demonstrated VTE safety in a meta-analysis analysing the effectiveness and safety of ustekinumab involving 13 studies in a UC setting (*n* = 1450), with only one incidence for DVT reported throughout. This meta-analysis assessed the effectiveness and safety in both subcutaneous and intravenous administrations [99].

Guselkumab, an IL-23 inhibitor, has limited, reported literature regarding VTE risk but has demonstrated comparable safety in its trials involving patients with moderative to severely active CD with inadequate response/intolerance to conventional therapy. The 48-week maintenance results of the phase two, GALAXI-1 trial indicate adverse event rates similar to placebo and ustekinumab groups, with no VTE events reported [100].

The literature for the upcoming Anti IL-12 and IL-23 therapies follows a similar trend, in that the data regarding VTE events in the IBD population are limited and further research is needed before any conclusive statement can be made on its increased risk for VTE.

## 7. Conclusions

This review highlights the significant association between IBD and VTE, elucidating the pathophysiological mechanisms and identifying key risk factors. The findings indicate that both UC and CD patients have an elevated risk of VTE, with UC patients particularly prone during pregnancy and the postpartum period.

Despite the clear evidence linking IBD with increased VTE risk, especially during disease flare-ups and post-discharge from hospital, there remains a notable gap in the literature regarding the long-term management and prophylactic strategies for VTE in this patient population.

There is a sparseness of studies specifically designed to investigate the VTE risk associated with common medications used to treat IBD. The available studies often have small sample sizes and are not always IBD-specific, making it difficult to draw definitive conclusions about medication-related VTE risk for patients with IBD. In addition, the differential risk between UC and CD underscores the need for more targeted research.

## 8. Future Directions

Future research should focus on large-scale, IBD-specific studies to better understand the relationship between IBD medications and VTE risk. In particular, JAKi therapies require much needed attention with regards to the dose-specific VTE risks in appropriately sized IBD populations. There is also a critical need for longitudinal studies to evaluate the effectiveness of various prophylactic strategies, particularly with respect to post-discharge risk scores and in pregnant women with IBD. Additionally, comparative studies involving other autoimmune diseases could provide valuable insights into the unique prothrombotic mechanisms in IBD. Common comorbidities such as diabetes mellitus and their interaction with an IBD population in assessing VTE risk is a literature gap, which should be a focus of further research. Another consideration of VTE risk not mentioned in this narrative review is the effect of physical activity in IBD populations. Exploration of the psychological effects that the diagnosis may exhibit on physical activity and its consequent effect on VTE pathogenesis has not been considered. Preliminary studies such as the BE-FIT-IBD [101] have raised attention towards this effect of physical activity and disease severity on IBD, but not specifically on VTE risk.

Addressing these research gaps will be essential in developing comprehensive guidelines to mitigate VTE risk and improve the overall management of IBD patients in different settings.

## Figures and Tables

**Table 1:** Summary table indicating the literature-reported perceived thrombotic effects of medications to treat an IBD population.

Drug Type/Name	Perceived Thrombotic Effect in IBD Populations
5-ASAs	Uncertain—no additional risk observed
Corticosteroids	Increased
Thiopurines	Uncertain—no additional risk observed
Methotrexate	Uncertain—no additional risk observed
TNF-a Inhibitors	Reduced
JAK inhibitors	Uncertain—drug and dose-dependent
S1P receptor modulators	Neutral
Anti-integrin	Uncertain—no additional risk observed
Anti-IL-12 and IL-23	Uncertain—no additional risk observed

## Data Availability

No new data were created or analysed in this study.

## Supplementary Materials

The following supporting information can be downloaded at: https://www.mdpi.com/article/10.3390/diagnostics14192112/s1; Table S1: Adaptation from RCOG regarding risk factors of VTE in pregnancy including IBD; Table S2: Adaptation from RCOG regarding risk factors of VTE in pregnancy, risk-stratification interpretation for what thromboprophylaxis is recommended in the antenatal and postnatal periods.
