# Supplementary material for: Controversies in Venous Thromboembolism Risk Assessment in Inflammatory Bowel Disease: A Narrative Review
Source: Diagnostics (Basel). 2024 Sep 24;14(19):2112. doi: 10.3390/diagnostics14192112 (PMC11476391; doi:10.3390/diagnostics14192112)
Supplement: Supplementary file 1 [file diagnostics-14-02112-s001.zip › diagnostics-3156670-supplementary.pdf]

Supplementary Materials

Supplementary Table S1: Adaptation from RCOG regarding risk factors of VTE in pregnancy including IBD

**Risk factors for VTE**	Score
**Pre-existing risk factors**	
Previous VTE (except single event related to major surgery	4
Known- high risk thrombophilia	3
Medical comorbidities including IBD	3
Obesity	1 (BMI>30)<br>2 (BMI>40)
Family history of unprovoked or oestrogen related VTE in 1st degree relative	1
Known-low risk thrombophilia (no VTE)	1
Age >35 years	1
Parity >/3	1
Smoker	1
Gross Varicose Veins	1
**Obstetric risk factors**	
Caesarean section in labour	2
Pre-eclampsia in current pregnancy	1
ART/IVF (antenatal only)	1
Multiple pregnancy	1
Elective caesarean section	1
Mid-cavity or rotational operative delivery	1
Prolonged labour (>24 hours)	1
PPH (>1 litre or transfusion)	1
Preterm birth <37 weeks in current pregnancy	1
Stillbirth in current pregnancy	1
**Transient risk factors**	
OHSS (1st trimester only)	4
Hyperemesis	3
Surgical procedure in pregnancy or puerperium except immediate repair of perineum	3
Current systemic infection	1
Immobility, dehydration	1

Abbreviations: VTE (Venous Thromboembolism), IBD (Inflammatory Bowel Disease), BMI (Body Mass Index), ART (Assisted Reproductive Technology), IVF (In Vitro Fertilisation), PPH (Postpartum Haemorrhage), OHSS (Ovarian Hyperstimulation Syndrome)

Supplementary Table S2: Adaptation from RCOG regarding risk factors of VTE in pregnancy, risk-stratification interpretation for what thromboprophylaxis is recommended in the antenatal and postnatal periods [25].

**Antenatally**
If total score ≥ 4= Consider thromboprophylaxis from the 1st trimester
If total score 3= Consider thromboprophylaxis from 28 weeks
If admitted to hospital antenatally= Consider thromboprophylaxis
**Postnatally**
If total score ≥ 2= Consider thromboprophylaxis for at least 10 days
· If readmission to hospital within puerperium (or prolonged admission ≥ 3 days)= Consider thromboprophylaxis
